# Influence of Graphene Oxide Concentration when Fabricating an Electrochemical Biosensor for DNA Detection

**DOI:** 10.3390/bios9040113

**Published:** 2019-09-26

**Authors:** Elena A. Chiticaru, Luisa Pilan, Celina-Maria Damian, Eugeniu Vasile, Jorge S. Burns, Mariana Ioniţă

**Affiliations:** 1 Faculty of Medical Engineering, University Politehnica of Bucharest, Gh Polizu 1-7, 011061 Bucharest, Romania; 2 Department of Inorganic Chemistry, Physical Chemistry and Electrochemistry, University Politehnica of Bucharest, 1-7, Polizu St., 011061 Bucharest, Romania; 3 Advanced Polymer Materials Group, University Politehnica of Bucharest, Gh Polizu 1-7, 011061 Bucharest, Romania; 4 Faculty of Applied Chemistry and Material Science, University Politehnica of Bucharest, 1-7 Gh. Polizu, 011061 Bucharest, Romania; 5 Laboratory of Cellular Therapies, Department of Medical and Surgical Sciences for Children & Adults, University Hospital of Modena and Reggio Emilia, 41121 Modena, Italy

**Keywords:** reduced graphene oxide, electrochemical biosensor, screen printed carbon electrodes, DNA adsorption

## Abstract

We have investigated the influence exerted by the concentration of graphene oxide (GO) dispersion as a modifier for screen printed carbon electrodes (SPCEs) on the fabrication of an electrochemical biosensor to detect DNA hybridization. A new pretreatment protocol for SPCEs, involving two successive steps in order to achieve a reproducible deposition of GO, is also proposed. Aqueous GO dispersions of different concentrations (0.05, 0.1, 0.15, and 0.2 mg/mL) were first drop-cast on the SPCE substrates and then electrochemically reduced. The electrochemical properties of the modified electrodes were investigated after each modification step by cyclic voltammetry (CV) and electrochemical impedance spectroscopy (EIS), while physicochemical characterization was performed by scanning electron microscopy (SEM), X-ray photoelectron spectroscopy (XPS), and Raman spectroscopy. Finally, the sensing platform was obtained by the simple adsorption of the single-stranded DNA probe onto the electrochemically reduced GO (RGO)-modified SPCEs under optimized conditions. The hybridization was achieved by incubating the functionalized SPCEs with complementary DNA target and detected by measuring the change in the electrochemical response of [Fe(CN)_6_]^3–/4–^ redox reporter in CV and EIS measurements induced by the release of the newly formed double-stranded DNA from the electrode surface. Our results showed that a higher GO concentration generated a more sensitive response towards DNA detection.

## 1. Introduction

Increased prevalence of genetic analysis fosters development of biosensors for rapid and cost-effective detection of specific DNA sequences within a broadening range of human diseases. Several approaches for DNA analysis systems such as optical (fluorescent, surface plasmon resonance luminescent, colorimetric, etc.) [1,2,3,4,5], piezoelectric [6], or electrochemical [7,8,9] have been proposed. The electrochemical approach is promising because it can provide the above advantages whilst also offering the possibility of self-powered miniaturized devices relevant for point-of-care applications [10,11,12].

An impedimetric DNA biosensor converts the biochemical affinity binding event of the target molecule with the DNA probe into an analytical signal corresponding to the charge-transfer resistance (R_ct_) between a redox-active species from the solution and the electrode surface. In contrast to classic approaches for DNA sensors, with target DNA labeled by an enzyme or a fluorophore, electrochemical impedance spectroscopy (EIS) offers label-free detection in addition to the advantages of relative simplicity, low cost, ease of miniaturization, and portability [13,14]. Other electrochemical detection methods include the common amperometric or voltammetric techniques, such as cyclic voltammetry (CV), differential pulse voltammetry and square-wave voltammetry, which involve the measurement of a current related to the analyte concentration under controlled potential conditions [15,16]. EIS is a technique of choice because it can detect significant changes in the signal in a low target concentration range [14,17] and is non-destructive, due to the low amplitude voltage perturbation that is much smaller than that used in amperometric testing [18,19,20,21].

Graphene has been studied for electrochemical sensing because of its outstanding properties, e.g., very high electrical conductivity, chemical stability, large surface-to-volume ratio, and excellent carrier mobility. However, this two-dimensional (2D) material is also highly hydrophobic, making it difficult for it to interact with biological molecules such as nucleic acids [22,23]. Graphene oxide (GO) and reduced graphene oxide (RGO) represent alternatives with oxygenated functionalities introduced into the carbon structure that enable dispersibility in water [24,25]. However oxygenated functionalities disrupt structural uniformity and compromise conductivity, so synthesis of RGO by various methods, e.g., thermal, chemical, or electrochemical approaches has aimed to achieve a balance between the highly conductive properties of pristine graphene and the functionalities of GO, retaining a sufficient number of electroactive sites on the surface to improve biosensor sensitivity [26,27,28]. DNA can be immobilized on graphenic surfaces by both covalent and non-covalent (physical adsorption) approaches. The main mechanism of DNA adsorption on RGO consists of the noncovalent π–π stacking between the aromatic rings of DNA nucleobases with hexagonal carbon rings forming the graphenic lattice, followed by hydrogen bonding and electrostatic repulsion between the oxygenated functional groups and oligonucleotides [29,30,31]. The adsorption of single-stranded (ss) DNA on graphene is reported to be efficient even in low ionic strength buffers, whereas double-stranded (ds) DNA, with its double-helical structure consisting of a negatively charged phosphate backbone surrounding the positively charged nucleobases, has a slower rate of adsorption [32,33,34,35]. This weaker interaction between RGO and dsDNA results in a quick desorption after hybridization of ssDNA probe with its complementary ssDNA target. Therefore, the high differentiation capacity of RGO between these configurations of DNA biomolecules has been frequently exploited in the design of electrochemical DNA biosensors [36,37,38,39,40].

Recently, screen-printed electrodes (SPEs) have been adopted for electroanalysis [41], favored over the traditional carbon electrodes (glassy carbon and carbon paste), because they are portable, compact, and inexpensive to manufacture, facilitating mass production of biosensors of high quality and reproducibility, suitable for point-of-care applications [42]. Even if SPEs and graphene have been extensively investigated, the large-scale production of such electrochemical detection platforms is rarely reported. The performance of an electrochemical biosensor for oligonucleotide hybridization can be strongly influenced by fabrication aspects.

In the latest years, several pretreatment procedures for the SPCE surface aiming to improve the sensitivity and reproducibility of the results have been reported [43,44,45,46,47,48,49,50]. Improvement of the electrochemical performance of screen-printed carbon electrodes by UV/ozone modification was very recently described [43]. The augmented electron transfer rate and the decreased peak-to-peak potential separation from 170 mV to 112 mV were mainly attributed to an increase in oxygen functional groups [43]. Nonetheless, due to its simplicity, the electrochemical pretreatment is often used for improving carbon electrode responses. This improvement was explained by a substantial removal of the organic binder, inducing a clear exposure of the micrometer-sized graphite particles at the electrode surface [49]. Several approaches for improving the voltammetric behavior of SPCEs, presented as activation or electrochemical cleaning procedures, consisted in repetitive cyclic voltammograms in various media: in diluted H_2_O_2_ from +1.0 to −0.7 V [46], in 0.5 M H_2_SO_4_ from −1 V to +1 V [48], in phosphate buffer solutions from −0.6 to +1.6 V [49], or only by applying anodic potentials ranging from +0.2 V to +2 V [47]. However, for all these electrochemical procedures, no reproducibility studies have been reported and the improvement in the response was presented either as a reduction of the semicircle in the Nyquist plot at the pre-treated SPCE [49] or as a narrower peak-to-peak separation (no ΔEp values) of the recorded voltammograms [46] for the reporter redox probes.

Among various transfer methods engineered for transfer of graphenic materials to enhance electrode performance, wet chemical methods have gained popularity by being relatively simple, convenient and scalable [51,52]. The most frequently encountered technique of employing SPEs for graphene investigation in electrochemistry requires modification of a graphite-based or carbon black-based electrode by drop-casting graphene or one of its derivatives on the SPE’s surface [53]. However, the technique can lead to the modified electrode having poor sensitivity and reproducibility, especially if all the modification steps are not carefully controlled and standardized. 

Another reported method to design graphene-based SPEs is the direct electrochemical reduction of the dispersed graphene oxide at the substrate electrode [54,55]. Although such electrochemical approaches can provide a more accurate control of the film thickness, they are poorly applicable to a large-scale production [16]. Inkjet-printing has been reported by Tuantranont et al. as a large-scale modifying approach for SPEs [56] that allows controllable dispersion deposition [57,58]. By modulating the number of printed layers, SPCEs modified with a graphene–poly(3,4-ethylenedioxythiophene):poly(styrenesulfonate) dispersion conducted to a satisfactory reproducibility for various analytes. However, the rheological properties of the inks frequently represent a major limitation of this strategy [16].

We here report a new, simplified, reproducible, and potentially scalable protocol for fabricating RGO-modified SPCEs (RGO-SPCEs). A preliminary two-step electrochemical treatment of commercially available SPCEs had a crucial effect on the electrochemical behavior of SPCEs and the reproducibility of the electrochemical signal. After pretreatment, the electrochemical properties such as peak-to-peak separation (ΔEp) and electron transfer resistance (Rct) of the ferri/ferrocyanide redox reporter in the solution were significantly improved. Moreover, the performance of these electrodes as a DNA hybridization detection platform could be simply modulated by varying the concentration of dispersed GO used for SPCE modification.

## 2. Materials and Methods

### 2.1. Reagents and Instrumentation

Graphene oxide (GO) in H_2_O (2 mg/mL), HCl, KCl, HNa_2_O_4_P, and H_2_NaO_4_P were procured from Sigma-Aldrich (St. Louis, MO, USA), while K_3_[Fe(CN)_6_] and K_4_[Fe(CN)_6_] × 3H_2_O were acquired from Merck Co., (Darmstadt, Germany). Single-stranded DNA probe (5’-TTT CAA CAT CAG TCT GAT AAG CTA TCT CCC-3’), its complementary single-stranded DNA target (5’-GGG AGA TAG CTT ATC AGA CTG ATG TTG AAA-3’), and IDTE buffer were obtained from Integrated DNA Technologies, Inc (Coralville, IA, USA). Before and after any modification, the electrodes were washed with ultrapure water (Adrona Crystal EX water purification system, 18.2 MΩ × cm resistivity).

Electrochemical measurements were performed at room temperature (~25 °C) with a potentiostat/galvanostat Autolab PGSTAT 204 (Metrohm Autolab, the Netherlands) controlled by NOVA 2.1 software. The experiments were carried out using a three-electrode system consisting of a screen-printed carbon electrode (SPCE—DRP 110 from DropSens, Spain) with 4 mm internal diameter as a working electrode (WE), a platinum wire auxiliary electrode, and a Ag/AgCl (3 M KCl) reference electrode in order to reference all potentials. The electrochemical cell containing all three electrodes was placed in a Faraday cage (Metrohm Autolab, the Netherlands) to shield the electrochemical system against electromagnetic interference. Electrochemical characterization of functionalized electrodes was done by cyclic voltammetry (CV) and electrochemical impedance spectroscopy (EIS). EIS measurements were carried out in the frequency range of 0.01 Hz to 100 kHz, at 10 mV AC amplitude, and an applied bias DC potential of +0.2 V, while in CV a scan rate of 0.05 V/s was applied. All electrochemical measurements were carried out in 1 mM K_3_[Fe(CN)_6_]/K_4_[Fe(CN)_6_] (1:1) redox probe in 0.1 M KCl solution. The impedance spectra obtained were represented as Nyquist plots in a complex plane and fitted by a Randles equivalent circuit.

The morphology of the modified electrodes was investigated by scanning electron microscopy (SEM), while the structural characterization was done by X-ray photoelectron spectroscopy (XPS) and Raman spectroscopy. SEM images were obtained with an electronic scanning microscope (SEM-QUANTA INSPECT F) by recording the resultant secondary electron beam with 30 keV energy. XPS analysis was performed on a K-Alpha spectrometer from Thermo Scientific (Waltham, MA, USA) equipped with a monochromated Al Kα source (1486.6 eV), operating in vacuum at a base pressure of 2 × 10^−9^ mbar. Flood gun compensated charging effects, and binding energies were calibrated by placing the C1s peak at 284.8 eV as an internal reference. Raman measurements were performed with a Renishaw inVia Raman confocal spectrometer, using a 473 nm laser excitation (Renishaw, Brno-Černovic, Czech Republic), the 100× objective, and 5% laser power.

### 2.2. Preparation and Testing of the Modified Electrodes

The pretreatment of SPCEs prior to GO modification consisted of five CV cycles from +0.5 to −1.5 V in 0.1 M HCl, followed by two CV cycles from 0 to +2 V in phosphate buffer solution (0.1 M PBS, pH 7) at a scan rate of 0.05 V/s. The CV response of the Fe(CN)_6_^3−/4−^ redox species at the pristine unmodified SPCE was poor, with a large peak separation of 195 ± 28 mV (*n* = 5 devices), indicating a slow electron transfer rate and irreversible electrochemical process. However, after the electrochemical pretreatment the difference between the peak potentials (ΔEp) was reduced to 93 ± 2 mV for all SPCEs. After this activation treatment, the electrodes were washed in ultrapure water (UPW), dried, and coated with PBS by very carefully dropping 1 µL solution on the whole surface of the working electrode without crossing the margin (this step proved to be very important in order to achieve a reproducible deposition of GO). After the solution dried on the carbon surface, the electrode was washed again with UPW then dried at 50 °C in the oven. Subsequently, 3 µL GO (0.05, 0.1, 0.15, and 0.2 mg/mL) was carefully dropped on the dry SPCE surface. After drying at room temperature for 2 hours, GO-modified SPCEs (GO-SPCEs) were electrochemically reduced by five CV cycles from 0 to −1.5 V, 0.05 V/s, in 0.5 M KCl, and then again dried at room temperature, and washed with UPW. In order to observe the electrode response after each stage of surface modification, CVs and impedimetric spectra were recorded in the presence of a Fe(CN)_6_^3−/4−^ redox system.

Finally, the ssDNA probe was immobilized by carefully dropping 6 µL DNA solution (10 µM) on the RGO-SPCEs. After drying at room temperature, the electrodes were washed with UPW in order to remove weakly adsorbed nucleotides at the RGO surface. Afterwards, the SPCEs were introduced in 30 µL DNA target solution (100 nM) to allow the formation of the hybridized dsDNA (at 58 °C for one hour)**.** If not used immediately, the electrodes were stored in air at 4 °C.

## 3. Results and Discussion

### 3.1. Morphological Characterization

**Figure 1:**
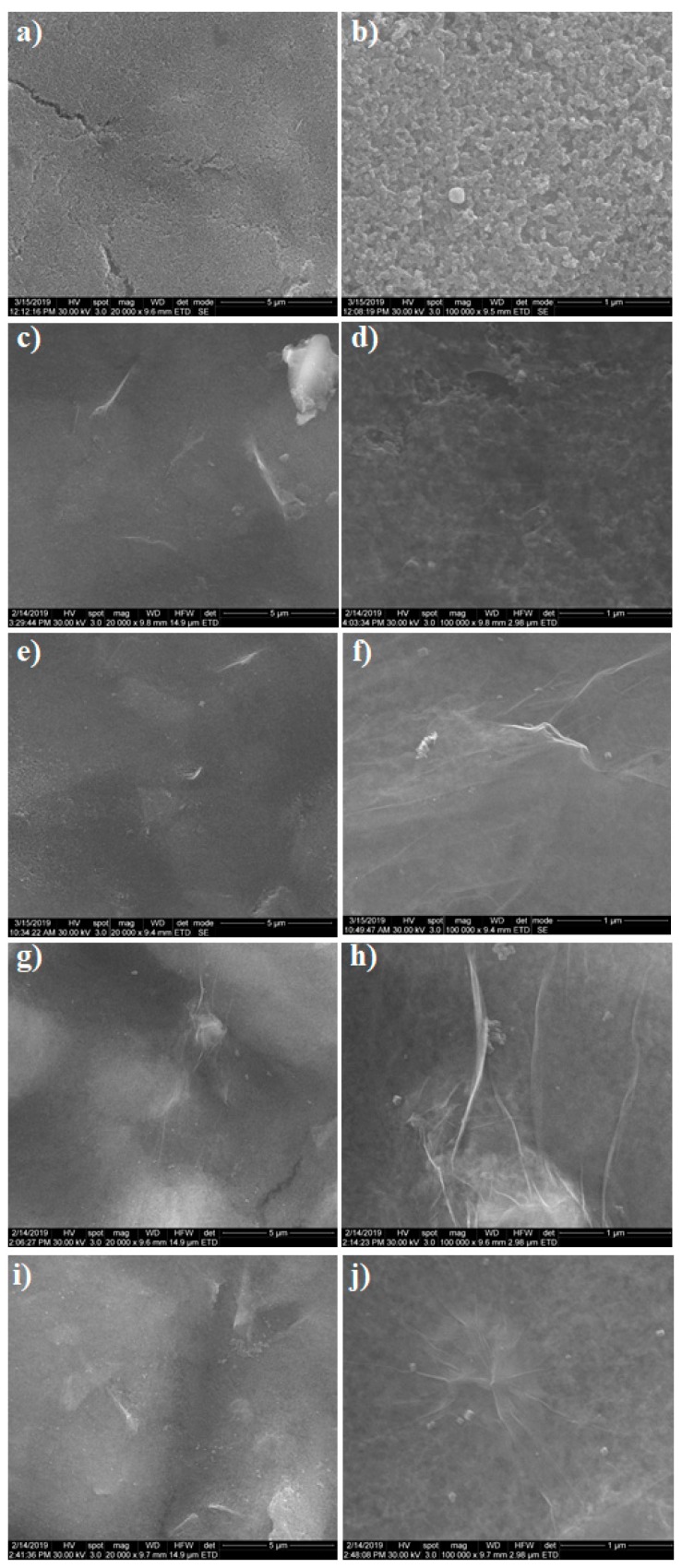
(**a**,**b**) SEM images of bare screen printed carbon electrode (SPCE). (**c**-**j**) SEM images of graphene oxide (GO) deposited on SPCE using different dispersion concentrations: (**c**,**d**) 0.05 mg/mL; (**e**,**f**) 0.1 mg/mL; (**g**,**h**) 0.15 mg/mL; and (**i**,**j**) 0.2 mg/mL. (**a,c,e,g,i**) Images recorded at 20 kX magnification. (**b,d,f,h,j**) Images recorded at 100 kX magnification.

Scanning electron microscopy was used to investigate the surface morphology of GO and to evaluate the homogeneity of the resultant GO-modified surface. Images were taken from three different spots on every electrode and the ones reflecting the most relevant characteristics were chosen. The pictures were taken at two different magnifications 20 kX (Figure 1a,c,e,g,i) and 100 kX (Figure 1b,d,f,h,j) in order to assess both surface homogeneity and morphology, respectively. Figure 1a,b shows the SEM images of the pretreated SPCE surface, while Figure 1c–j is characteristic of the GO-SPCE-modified electrodes with different GO dispersion concentrations. A clear resemblance of the images corresponding to 0.05 mg/mL GO (Figure 1c,d) and 0.1 mg/mL GO (Figure 1e,f) was observed, in such cases the electrodes surface was predominantly covered with thin smooth sheets of graphene, and the carbon substrate could still be observed. Likewise, the images corresponding to 0.15 mg/mL GO (Figure 1g,h) and 0.2 mg/mL GO (Figure 1i,j) shared similar graphene-like morphology, showing wrinkled sheets with folded margins and seldomly agglomerated GO flakes. Finally, it was important to notice a homogeneous coverage on all four electrodes and an increased thickness of GO layers with the increase of the dispersion concentration.

**Figure 2:**
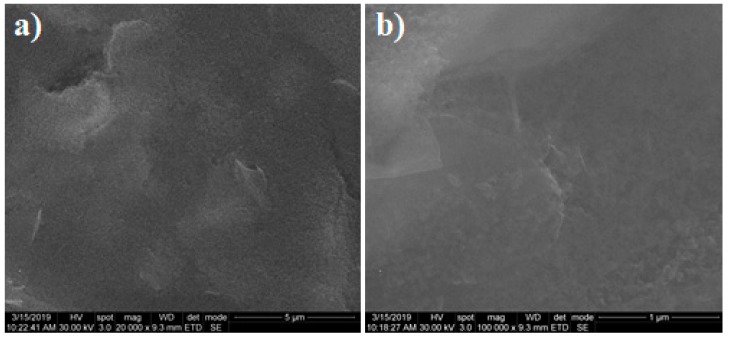
SEM images of reduced graphene oxide (RGO)-modified SPCE using 0.15 mg/mL dispersion recorded at (**a**) 20 kX and (**b**) 100 kX magnifications.

SEM images were also recorded after the electrochemical reduction of graphene oxide. Figure 2 shows the characteristics of 0.15 mg/mL RGO obtained at 20 kX (Figure 2a) and 100 kX (Figure 2b) magnifications. The reduced form of GO maintained a similar morphology, showing a good coverage of the electrode with thin and slightly wrinkled layers that had a tendency to agglomerate. Similar characteristics were obtained for the other three concentrations of RGO-modified electrodes. 

### 3.2. Structural Characterization

XPS provided an ultimate analysis of GO and RGO surface elemental composition. The survey spectra of all GO-based samples showed mainly the presence of carbon and oxygen with trace amount of nitrogen, which was attributed to process contamination. The reference used to evaluate the presence of oxygenated functional groups from the GO sheets was the C to O atomic ratio from XPS survey spectra. Based on this assessment it was observed that the GO samples had a very high oxygen atomic percentage (C/O ratio value close to 3). After electrochemical reduction there was a significant decrease in the signals for oxygen-containing functional groups, indicative of an efficient reduction of GO to RGO. Notably, the atomic ratio C/O was increased to a value of almost 6, indicating that the delocalized π conjugation was to some extent restored in the RGO samples. This composition ratio was also calculated for a DNA sample, in order to assess its contribution to the decrease in C/O ratio after DNA immobilization. Thus, it was shown that a 1.67 value for C/O ratio could slightly influence the overall atomic mapping of the RGO + DNA sensor by giving an increased C content. 

Regarding the nitrogen and phosphorous, the sensors containing immobilized DNA showed increased P content reaching a maximum for the 0.15 mg/mL sample (Table 1).

A deeper analysis of the chemical state of C element was acquired through the deconvolution of a high-resolution C1s XPS spectrum. The GO-based electrodes showed a sharp peak at 284.5 eV that corresponded to carbon atoms involved in C–C bonds, coming from the conjugated honey-comb lattice. The peaks observed at 285.1, 286.7, and 288.5 eV could be assigned to C–H species with sp^3^ hybridization, C–O, and C=O bonding configurations, respectively, due to the oxidation and destruction of the sp^2^ atomic structure of graphite. 

**Figure 3:**
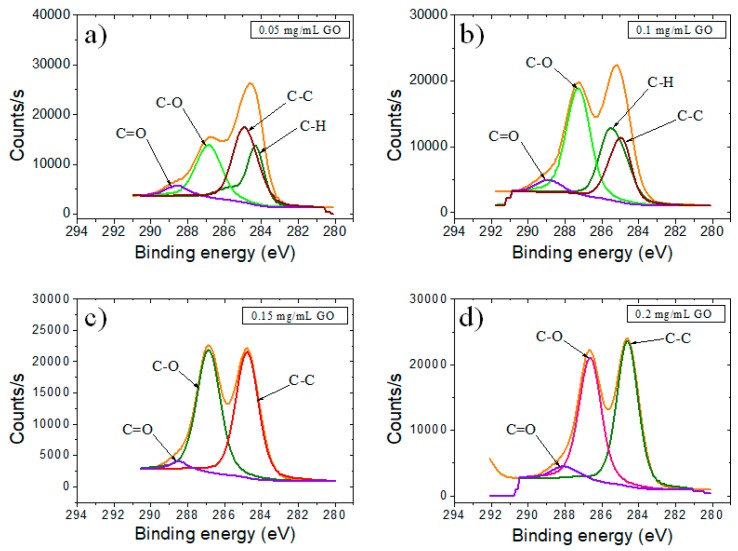
High-resolution C1 XPS spectra of SPCE modified with (**a**) 0.05 mg/mL GO; (**b**) 0.1 mg/mL GO; (**c**) 0.15 mg/mL GO; and (**d**) 0.2 mg/mL GO.

Comparing samples with different concentrations of GO (Figure 3), we observed a modification of the area corresponding to the 286.7 eV band from C1s high-resolution spectra assigned to C–O species. Generally, the peak area for C–O decreased with the decrease of GO concentration; however, we observed a maximum of this peak area for the samples corresponding to 0.15 mg/mL, slightly higher than the one corresponding to 0.2 mg/mL. This trend can be assigned to the stacking of the GO layers after reaching a threshold limit value. 

**Figure 4:**
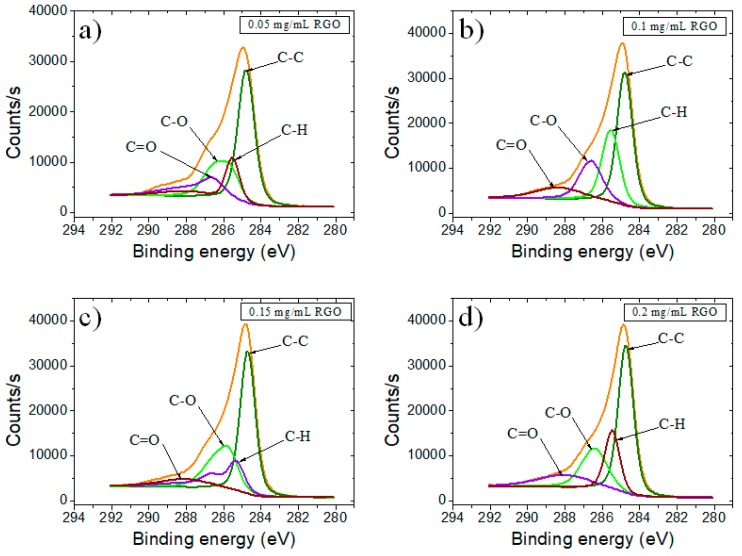
High-resolution C1 XPS spectra of SPCE modified with RGO using as precursor (**a**) 0.05 mg/mL GO; (**b**) 0.1 mg/mL GO; (**c**) 0.15 mg/mL GO; and (**d**) 0.2 mg/mL GO.

When GO was electrochemically reduced to RGO (Figure 4), it was observed a clear loss of oxygen moieties, that probably generated new sp^2^ C–C bonds. Accordingly, we observed an increased intensity of C–C species. However, the remaining epoxy, hydroxyl and carboxylic groups translated through the presence of secondary peaks from 286 and 288 eV would make aqueously dispersed RGO negatively charged, allowing a strong noncovalent binding between RGO and the nucleobases of ssDNA.

**Figure 5:**
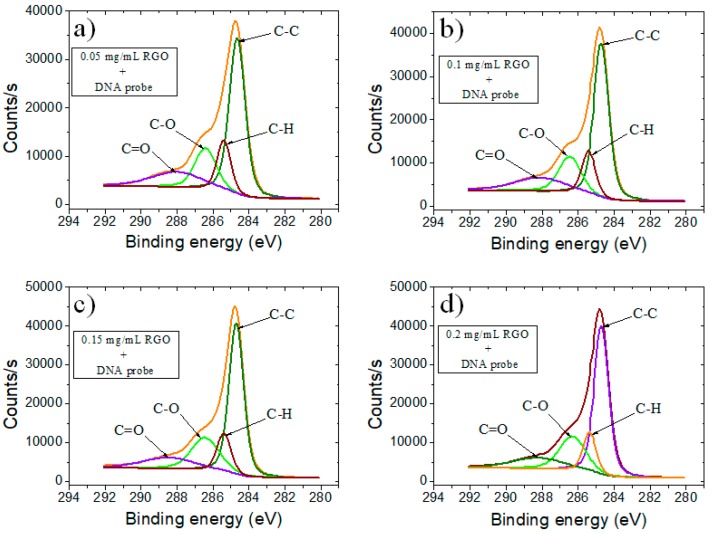
High-resolution C1 XPS spectra of 10 µm DNA probe immobilized on the surface of SPCE modified with RGO using as precursor (**a**) 0.05 mg/mL GO; (**b**) 0.1 mg/mL GO; (**c**) 0.15 mg/mL GO; and (**d**) 0.2 mg/mL GO.

When ssDNA was deposited on RGO (Figure 5), besides the presence of an increased N and P content in the wide range spectra, the clear shifting of the peak from 286.4 eV, that is related to the increase in the content of C–N species from ssDNA, provided further evidence of the immobilization. Nonetheless, there was no notable difference between intensities of heteroatom secondary peaks (C–O, C–N, and C=O respectively).

From Table 2 it can be noticed that physical interactions between ssDNA and RGO layers determined a shift to higher binding energies for C–O and C=O secondary C1s peaks from 286.2 to 286.4 eV and from 287.8 to 288.1 eV, respectively. Moreover, for each concentration the secondary C1s peaks were at the same position, meaning that in each case efficient interactions were formed.

**Figure 6:**
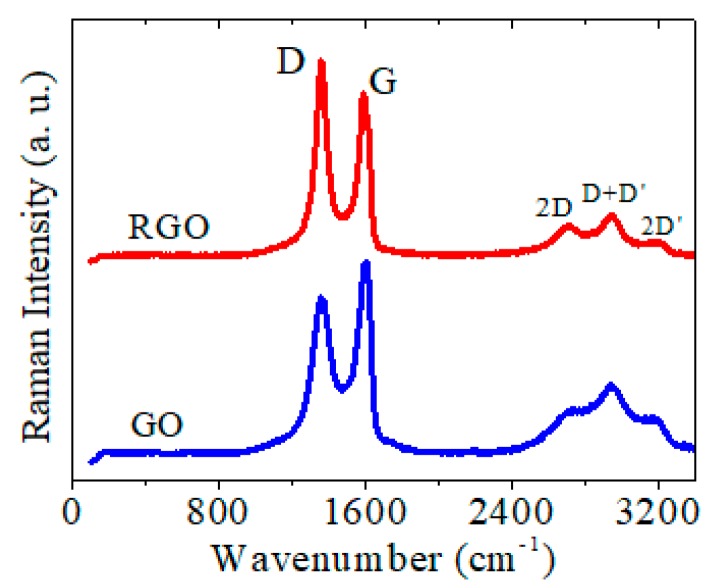
Raman spectra of GO and RGO (obtained by electrochemical reduction of GO films by five CV cycles from 0 to −1.5 V, 0.05 V/s, in 0.5 M KCl).

Raman spectroscopy was used to explore any graphenic structural changes after the electrochemical reduction. The GO characteristic D vibrational band at 1355 cm^−1^ and G band at 1601 cm^−1^ shifted for RGO, showing a D band at 1360 cm^−1^ and a G band at 1587 cm^−1^ (Figure 6). Besides this slight spectrum shift, the reduction of GO caused an increase in the D peak intensity (I_D_) and consequently the I_D_/I_G_ ratio increased from 0.8 to 1.15. These results indicated an increased defect concentration in the structure of RGO relative to that of GO, that meant a decreased average size of sp^2^ domains upon GO reduction, in agreement with the previous reports which showed that new smaller sized graphitic domains were created upon GO reduction [59,60].

### 3.3. Electrochemical Characterization 

Different GO concentrations were applied to the commercially available SPCEs to determine that most suited for the fabrication of a DNA hybridization biosensor. Measurement reproducibility was critically improved when a preliminary two step electrochemical treatment was applied to as-received commercial SPCEs. The pretreatment consisted of five repetitive voltammetric cycles from +0.5 to −1.5 V in 0.1 M HCl, followed by two cycles from 0 to +2 V in 0.1 M PBS, pH 7, at a scan rate of 0.05 V/s. Such activating treatment improved the electrochemical behavior of SPCEs such as peak-to-peak separation and electron transfer resistance (Rct) for the redox couple in the solution, and provided a hydrophilic carbon surface, facilitating the further deposition and adhesion of GO. Moreover, the addition of a small volume of PBS on the electrode could change the wetting properties of the surface to prevent spread of the GO beyond the outside border of the SPCE. This additional step proved to be essential for the GO coating reproducibility. Our studies showed that the 3 µL solution volume sufficed to obtain a graphenic film of consistent thickness and reproducible electrochemical signal.

**Figure 7:**
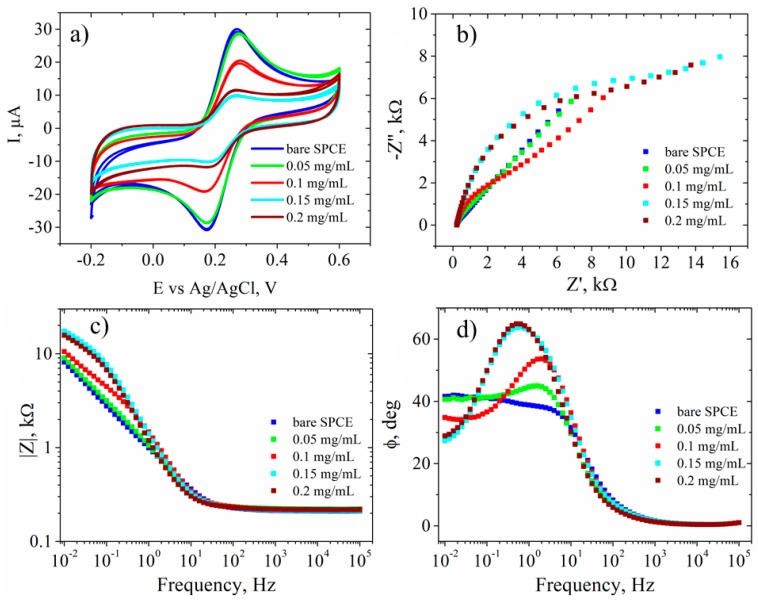
Electrochemical characterization: (**a**) CV; (**b**) EIS Nyquist plot; (**c**) EIS Bode plot—impedance modulus; (**d**) and phase shift. Characterizations were recorded in 1 mM [Fe(CN)_6_]^3−/4−^, 0.1 M KCl, for bare SPCE (blue) and GO-SPCEs obtained using different concentrations of GO dispersions: 0.05 mg/mL (green), 0.1 mg/mL (red), 0.15 mg/mL (cyan), and 0.2 mg/mL (dark red).

The electrochemical characterization in the presence of 1 mM [Fe(CN)_6_]^3−/4−^ as a redox couple (Figure 7) showed no change in the current intensity (Figure 7a) or in the Rct values (Figure 7b) for the 0.05 mg/mL GO-SPCE-modified electrode compared to the bare electrode. At 0.1 mg/mL GO a decrease of redox peaks (Figure 7a) and a correlated increase of the Rct (Figure 7b) was observed. Furthermore, the results show similar CV signals (Figure 7a) and EIS spectra (Figure 7b,d) for 0.15 mg/mL and 0.2 mg/mL GO, respectively. Such behavior at the latter GO-SPCEs, consisting in lower [Fe(CN)_6_]^3−/4−^ redox currents and increased Rct, could be envisioned as due to both the electrically insulating property of GO film and the repulsion of ferri/ferrocyanide ions with negatively charged functional groups of GO sheets.

**Figure 8:**
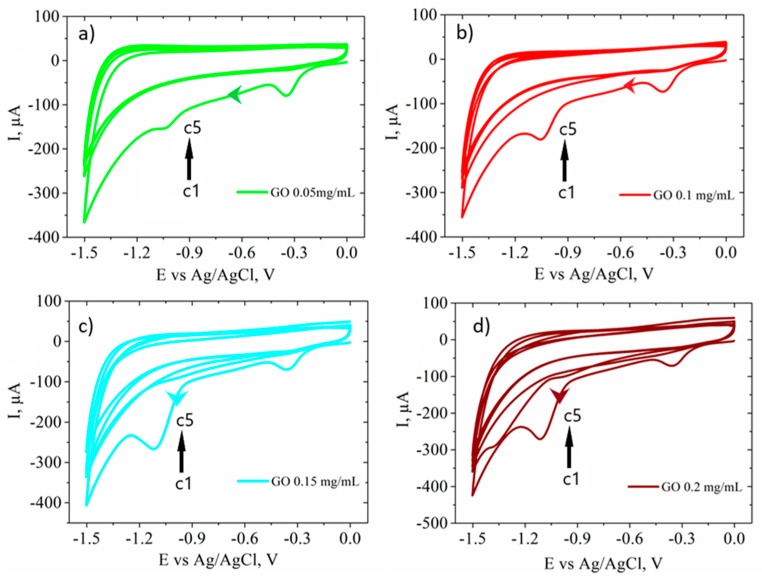
CVs (cycles (c) 1 to 5) showing the electrochemical reduction in 0.5 M KCl of GO-SPCEs obtained for different concentrations of GO dispersions: (**a**) 0.05 mg/mL; (**b**) 0.1 mg/mL; (**c**) 0.15 mg/mL; and (**d**) 0.2 mg/mL.

In the next step of biosensor fabrication, GO films were electrochemically reduced by potential cycling between 0 and −1.5 V (0.05 V/s) in 0.5 M KCl aqueous solutions and the corresponding CVs are illustrated in Figure 8. In the first potential cycle, a similar GO reduction peak was clearly distinguishable at approximately −0.3 V for all four samples. Also, a second reduction peak was observed between −1.0 V and −1.1 V in all plots, less evident when using 0.05 mg/mL GO (Figure 8a), but incrementally increased for higher GO dispersion concentrations. In the subsequent cycles the currents were diminished and the reduction peaks no longer observed, similar to other previously reported studies on the electrochemical reduction of GO [61]. A slight increase in the capacitive current with cycling was also observed due to an increased electrode active area from the gradually obtained RGO film. This behavior was more obvious when using 0.15 mg/mL and 0.2 mg/mL GO dispersions.

**Figure 9:**
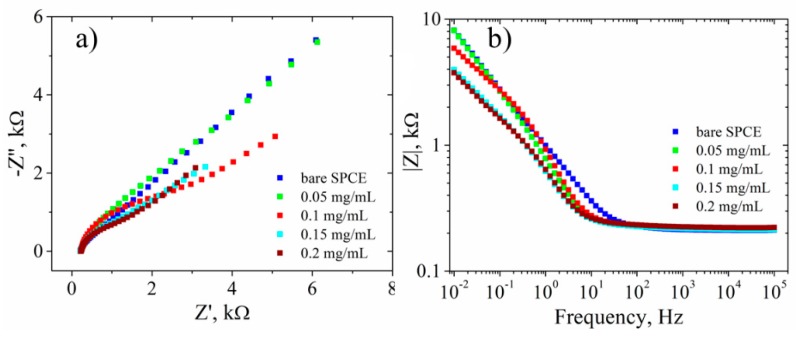
EIS characterization: (**a**) Nyquist plot; (**b**) EIS Bode plot—impedance modulus. Characterizations were recorded in 1 mM [Fe(CN)_6_]^3−/4−^, 0.1 M KCl, for bare SPCE (blue) and RGO-SPCEs obtained using different concentrations of GO dispersions 0.05 mg/mL (green), 0.1 mg/mL (red), 0.15 mg/mL (cyan), and 0.2 mg/mL (dark red).

After the electrochemical reduction of GO to RGO sheets of higher conductivity and neutral structure, the kinetics of the redox probe at the GO-SPCE was significantly improved (accelerated electron transfer evidenced by a decreased Rct, lower phase shift and smaller impedance of the system). However, such characteristics were only clearly observed for 0.15 mg/mL and 0.2 mg/mL GO samples, while no substantial changes occurred for 0.05 mg/mL sample compared to bare SPCE (Figure 9).

### 3.4. Electrode Response towards DNA Hybridization 

**Figure 10:**
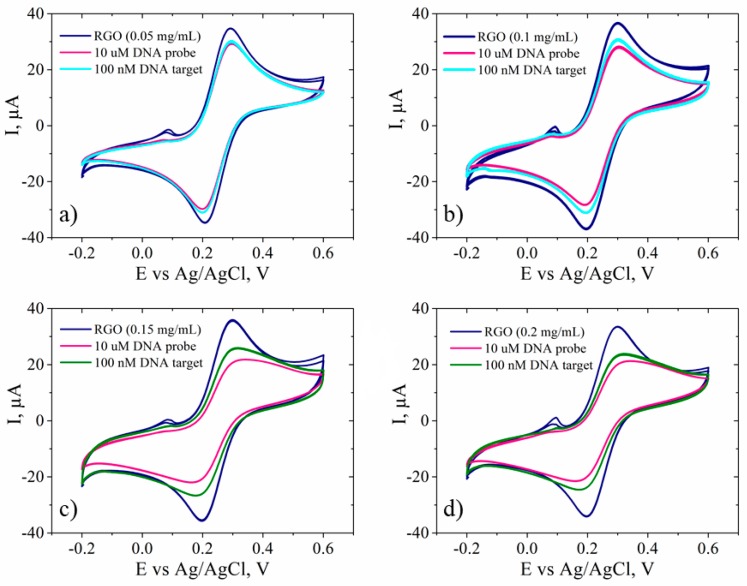
CV measurements recorded in 1 mM [Fe(CN)_6_]^3−/4−^, 0.1 M KCl, for RGO-SPCEs based on (**a**) 0.05 mg/mL; (**b**) 0.1 mg/mL; (**c**) 0.15 mg/mL; and (**d**) 0.2 mg/mL GO dispersions, after adsorption of ssDNA probe (10 µM) and hybridization with 100 nM DNA target.

The same electrochemical tools were used to detect changes following the functionalization of the RGO electrodes with DNA probe and hybridization with the complementary DNA target. After immobilization of ssDNA probe on the surface of RGO-SPCEs, significant changes in the peak current’s intensity and Rct of the ferri/ferrocyanide redox-active species from the test electrolyte were achieved for the samples that used higher GO concentrations, such as 0.15 mg/mL and 0.2 mg/mL rather than 0.05 mg/mL and 0.1 mg/mL (Figure 10). This behavior could be explained by the fact that immobilized ssDNA with a negatively charged phosphate backbone imparted an electrostatic repulsive force to [Fe(CN)_6_]^3−/4−^. Moreover, an increased thickness of the RGO film (obtained using higher initial GO concentrations) enhanced the accumulation of immobilized ssDNA probe, that could eventually lead to an improved sensitivity of the biosensor. An enhanced signal change, and thus a higher sensitivity, were achieved for the samples that used higher GO concentrations. The included SEM images indicated an increased thickness of GO layers with the increase of the dispersion concentration. Such behavior could be explained by the fact that an enhanced signal change indicated DNA hybridization, while minimal signal change indicated less binding. This suggested that more complementary ssDNA probe was adsorbed on 0.15 mg/mL and 0.2 mg/mL GO-based electrodes, because they exhibited significant response while use of other GO concentrations showed low or no change in signal.

**Figure 11:**
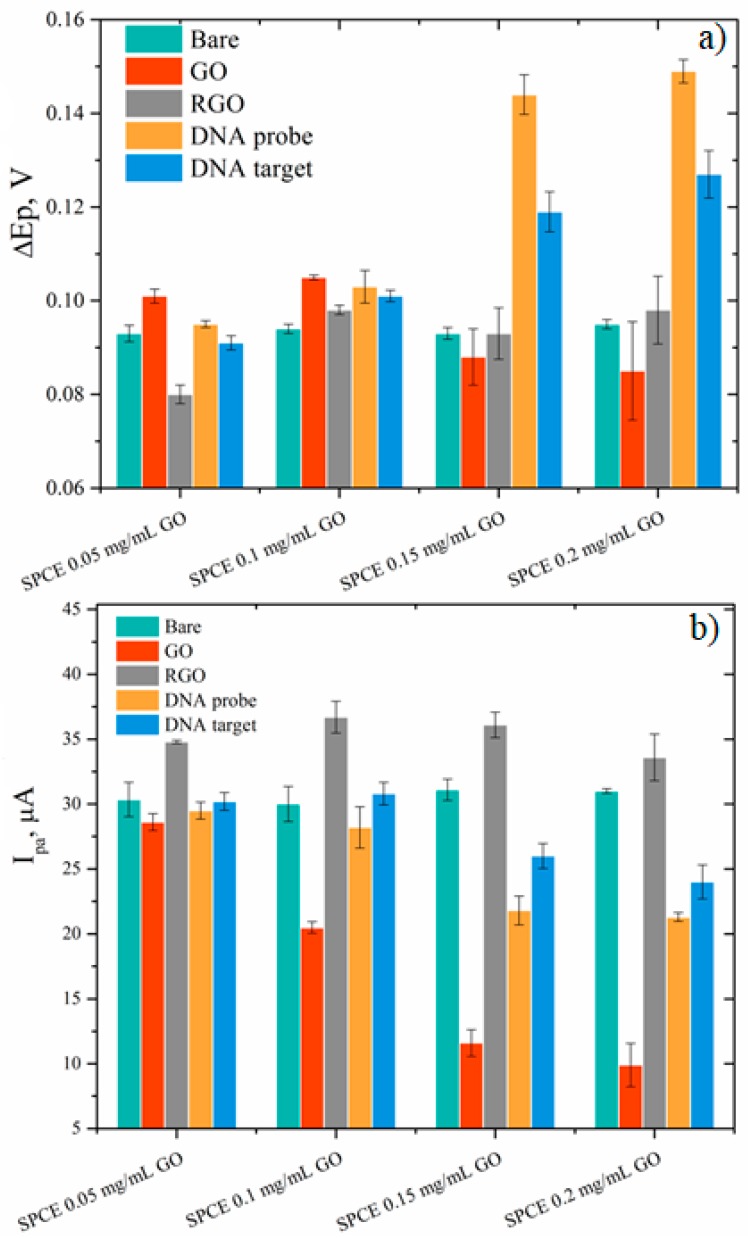
(**a**) Peak separation values (ΔE_p_) (**b**) and anodic peak currents (I_pa_) resulting from CV measurements recorded in 1 mM [Fe(CN)_6_]^3−/4−^, 0.1 M KCl, after every modification stage of SPCEs electrodes: drop-casting of GO dispersions of different concentrations (GO), electrochemical reduction of GO (RGO), adsorption of ssDNA probe (10 µM) on RGO-SPCEs (DNA probe) and hybridization with 100 nM target DNA (DNA target). Error bars represent standard deviation with *n* = 3.

**Figure 12:**
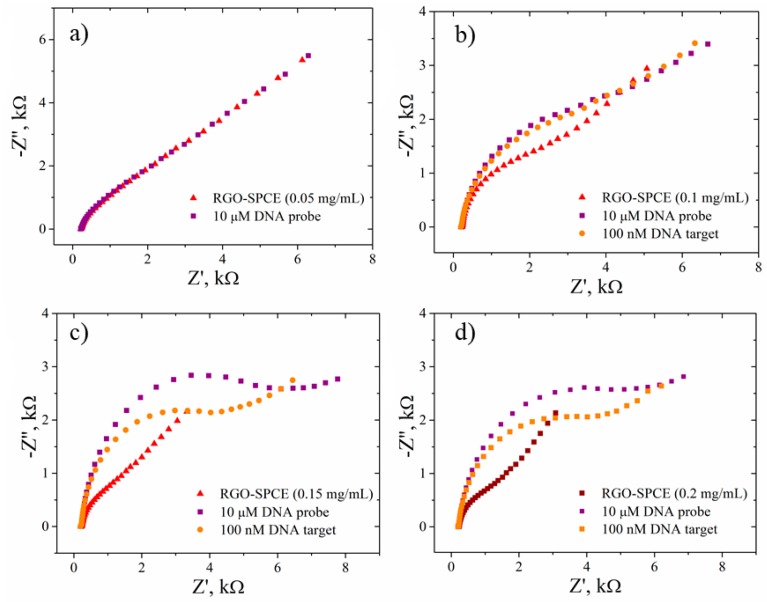
EIS measurements (Nyquist spectra) recorded in 1 mM [Fe(CN)_6_]^3−/4−^, 0.1 M KCl, for RGO-SPCEs based on (**a**) 0.05 mg/mL; (**b**) 0.1 mg/mL; (**c**) 0.15 mg/mL; and (**d**) 0.2 mg/mL GO dispersions, after adsorption of ssDNA probe (10 µM) and hybridization with 100 nM DNA target.

**Figure 13:**
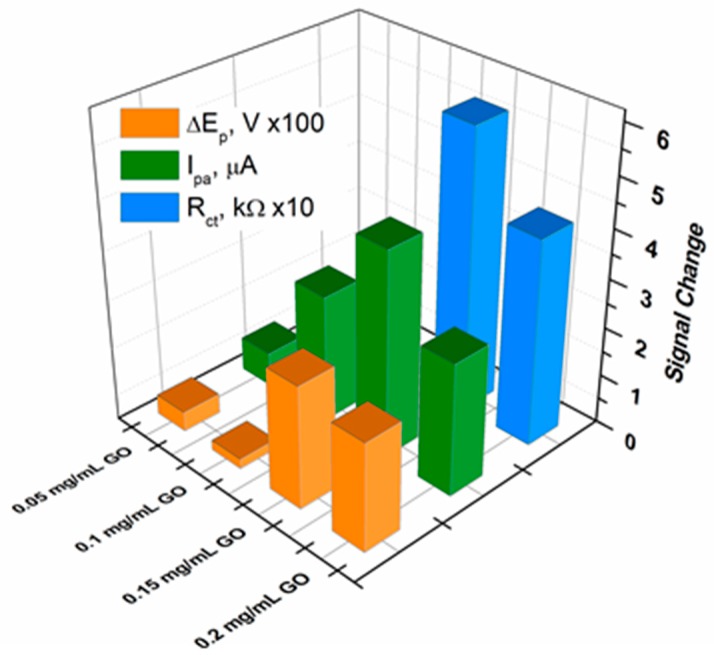
Signal change in peak current intensities (Ipa), peak-to-peak separation potentials (ΔEp) and charge transfer resistance values obtained from EIS measurements (Rct, quality of fitting χ^2^ = 0.051 ± 0.022) after hybridization with 100 nM DNA target in respect with the measured values after ssDNA probe adsorption.

The hybridization reaction was carried out by incubating ssDNA/RGO-SPCE functionalized electrodes in 30 µL DNA target (100 nM) at 58 °C for one hour. The subsequent characterization of the hybridized electrodes by CV in the presence of the redox probes showed, with the exception of only the 0.05 mg/mL sample, an increase of the peak currents and a reduction in peak-to-peak separation (Figure 10). This effect stemming from a more favorable kinetics for [Fe(CN)_6_]^3−/4−^ is more clearly evidenced by the chart in Figure 11, where we provide a complete comparison of the redox species response in CV after each modification stage of the electrodes. Since the ssDNA was noncovalently adsorbed to the RGO-SPCEs, hybridization with the complementary oligonucleotide sequence readily induced desorption of the dsDNA target-conjugated probes from RGO surfaces [37,40,62], favoring the redox reaction of [Fe(CN)_6_]^3−/4–^. Hence, the Rct values should decrease upon dsDNA formation, and such a sensitive impedimetric signal was achieved when using 0.15 mg/mL and 0.2 mg/mL GO samples (Figure 12 and Figure 13). 

Once again, it was obvious that for the samples prepared with low concentrations of GO dispersion, the changes in the electrochemical signal upon DNA hybridization were not as substantial as those observed for 0.15 mg/mL and 0.2 mg/mL GO. Moreover, at a very careful analysis of the results obtained for these most favorable GO concentrations, one can observe a better sensitivity in DNA target detection for the electrode prepared with 0.15 mg/mL GO (Figure 13). This outcome correlated very well with the XPS result that suggested a maximal ssDNA probe immobilization for the same sample.

## 4. Conclusions

This work presents a study on the influence induced by the concentration of GO dispersion as a modifier on commercially available SPCEs for developing RGO-SPCEs for electrochemical detection of DNA hybridization. 

A new and simple protocol consisting of two-step electrochemical treatment of the SPCEs was also proposed in order to achieve a reproducible deposition of GO on SPCEs. The pretreatment consisted of five repetitive voltammetric cycles from +0.5 to −1.5 V in 0.1 M HCl, followed by two cycles from 0 to +2 V in 0.1 M PBS, pH 7, at a scan rate of 0.05 V/s. This process substantially improved the electrochemical properties of the electrodes and facilitated the further deposition and adhesion of GO. Furthermore, an additional step of casting a small volume of PBS on the electrode to control the wetting properties of the surface proved to be essential for the coating reproducibility. 

This novel procedure for SPCE pretreatment would be broadly applicable to the fabrication of many types of disposable biosensors. The reproducibility of the deposited film could allow the scaling of the method to fabricate electrochemical sensing platforms with larger surface areas than those produced by conventional drop-casting, but further studies demonstrating such feasibility are needed.

Signal amplification is an important parameter for DNA hybridization biosensors, and we have demonstrated that the sensitivity of the RGO-SPCEs as DNA hybridization detection platform could be tuned by varying the concentration of aqueously dispersed GO used for SPCE modification. Best results in terms of sensitivity were obtained using 0.15 mg/mL GO.

Our work represents a proof-of-concept approach for fabricating reproducible screen-printed RGO electrodes that are paving the way for large-scale graphene-based sensing platforms in the future.

## Figures and Tables

**Table 1:** Carbon to oxygen ratio, nitrogen and phosphorous content of electrodes modified with four different concentrations of GO, after electrochemical reduction and after immobilization of ssDNA probe on the surface (values for DNA probe: C/O, 1.67; N(%), 10.02; P(%), 1.01).

Sample		GO	RGO	RGO + DNA probe
**0.05 mg/mL**	**C/O**	3.599	5.588	6.551
	**N(%)**	2.57	3.14	2.83
	**P(%)**	0	0	0.51
**0.1 mg/mL**	**C/O**	2.983	5.646	6.692
	**N(%)**	1.31	2.33	3.06
	**P(%)**	0	0	0.6
**0.15 mg/mL**	**C/O**	2.823	5.654	6.724
	**N(%)**	2.06	2.17	3.13
	**P(%)**	0	0	1.04
**0.2 mg/mL**	**C/O**	2.739	5.88	7.23
	**N(%)**	2.06	2.46	3.05
	**P(%)**	0	0	0.3

**Table 2:** Binding energies of specific bonds existing in graphene oxide, reduced graphene oxide, DNA probe immobilized on RGO, and DNA probe on a plastic substrate (values for DNA probe: C–C, 284.7; C–O, 286.2; C=O, 287.8).

Sample		GO	RGO	RGO + DNA probe
**0.05 mg/mL**	**C–C**	285.4	284.8	284.7
	**C–H**	285.1	285.5	285.4
	**C–O**	286.8	286.6	286.4
	**C=O**	288.5	288.2	288.1
**0.1 mg/mL**	**C–C**	284.8	284.8	284.7
	**C–H**	285.4	285.5	285.4
	**C–O**	287.1	286.6	286.4
	**C=O**	288.6	288.2	288.1
**0.15 mg/mL**	**C–C**	284.7	284.8	284.7
	**C–H**	-	285.6	285.4
	**C–O**	286.8	286.6	286.4
	**C=O**	288.5	288.1	288.3
**0.2 mg/mL**	**C–C**	284.7	284.8	284.7
	**C–H**	-	285.5	285.4
	**C–O**	286.7	286.5	286.2
	**C=O**	288.3	288.1	288.4
